# Zinc: An Emerging Axis of Host–Pathogen Interaction in Tuberculosis

**DOI:** 10.3390/vaccines14070567

**Published:** 2026-06-27

**Authors:** Jordan Holl, Jamie Corro, Bibhuti B. Mishra, Anil K. Ojha

**Affiliations:** 1 Division of Genetics, Wadsworth Center, New York State Department of Health, Albany, NY 12208, USA; jordan.holl@health.ny.gov (J.H.); jamie.corro@health.ny.gov (J.C.); 2 American Public Health Laboratories, Wadsworth Center, New York State Department of Health, Albany, NY 12208, USA; 3 Department of Immunology and Microbial Disease, Albany Medical College, Albany, NY 12208, USA; mishrab@amc.edu; 4 Department of Biomedical Sciences, University at Albany, Albany, NY 12208, USA

**Keywords:** zinc, neutrophils, nutritional immunity, calprotectin, S100A8/A9

## Abstract

Zinc is an essential micronutrient required by all forms of life, including *Mycobacterium tuberculosis* (Mtb), the etiological agent of tuberculosis. Mtb can persist within the host for years and requires a prolonged, multidrug treatment regimen for effective clearance. The sequestration of essential metals, including zinc, during bacterial infection is a key component of the host’s innate immune response. In this process, metal-chelating proteins such as the neutrophil-derived protein, calprotectin, play a central role in nutritional immunity by limiting microbial access to critical metal cofactors. Despite its importance, the impact of nutritional immunity on Mtb pathogenesis remains incompletely understood. In this review, we summarize recent advances in our understanding of zinc-responsive adaptations in Mtb and propose that zinc limitation within the host contributes significantly to the long-term persistence of this pathogen.

## 1. Introduction

The World Health Organization estimates that approximately one quarter of the global population is infected with *Mycobacterium tuberculosis* (Mtb), the causative agent of tuberculosis (TB) [1]. According to the Global Tuberculosis Report 2025, there were approximately 10.7 million cases of tuberculosis in 2024, with 1.23 million deaths [1]. One of the reasons for the staggering impact of TB on global public health is the extraordinary ability of Mtb to persist for months during chemotherapy, often without any clinical symptoms of disease [2,3]. A typical treatment regimen for tuberculosis entails daily administration of at least three antibiotics for 4–6 months [4]. Although bacterial load in the sputum appears to be reduced by over 90% within the first two weeks [5], and sputum conversion in the majority usually occurs by two months, an early withdrawal of antibiotics often leads to a high frequency of relapses [6,7]. Moreover, bacilli recovered from relapsed TB sputa have the same minimal inhibitory concentrations for the tested drugs as the strains cultured before the treatment [8], suggesting that the antibiotic tolerance of Mtb in the relapsed cases is an outcome of phenotypic variation across individual bacilli. Drug-tolerant subpopulations of Mtb cells have also been observed in multiple in vitro conditions, and are generally correlated with slow-growth and metabolic quiescence [9]. While the evidence for metabolically quiescent bacilli in hosts remains elusive, the two most extensively studied host-induced growth-restrictive conditions are low pH and hypoxia [10,11,12,13,14,15,16,17,18]. The acidification of phagolysosomes in Mtb-infected macrophages has been shown to restrict the growth of intracellular bacilli [19,20], thus supporting the hypothesis that the persister subpopulation could be harbored within phagolysosomes. Similarly, the obligate requirement of oxygen for Mtb growth and the presence of a hypoxic environment in TB granulomas together form the basis for host-induced hypoxic conditions as a key driver for the development of non-replicating drug-tolerant persisters in vivo [12,18]. However, the causal effect of low pH and/or hypoxia on the development of non-replicating persisters in vivo is yet to be demonstrated.

Recent findings from our studies and others reveal that the level of free zinc, an essential elemental micronutrient for all forms of life, is a crucial determinant of transcriptional and metabolic reprogramming in Mtb and other mycobacteria [21,22,23,24,25,26,27,28]. For example, a relatively modest decrease in the intracellular free zinc levels induces changes in the composition of the Mtb ribosome, which eventually undergoes hibernation upon further zinc depletion [25,26]. Ribosome hibernation is a highly conserved process across bacterial species, presumably to preserve the non-translating ribosomes in slow/non-replicating bacilli and enable them to restart protein synthesis during growth resuscitation [29]. Moreover, the evidence of zinc-limiting conditions in Mtb-infected host lungs [23,26,30], likely induced by the host innate immune system through a process commonly called nutritional immunity, raises the hypothesis that host sequestration of zinc from Mtb underlies the development of non-replicating persisters. Here, we present a literature review supporting this hypothesis.

### 1.1. Zinc: An Essential Elemental Nutrient for All Forms of Life, Including Mtb

Zinc is a vital micronutrient and is essential for various metabolic and enzymatic functions across all life forms, including DNA polymerase, RNA polymerase, tRNA synthetases, and enzymes responding to oxidative stress [31]. In prokaryotes, it is estimated that zinc is bound to approximately 5–6% of cellular proteins, with roughly 50% identified as catalytic enzymes, and 20% requiring zinc as a structural component [32,33,34]. In addition to structural and catalytic roles, zinc acts as a regulatory signal that modulates gene expression and stress responses in bacteria [31,35]. Although the total intracellular concentration of zinc ranges from 0.1 to 1 mM, the majority of zinc is closely associated with various metalloproteins, while the pool of free zinc ions remains limited to the picomolar range [32,35,36]. The essential role of zinc in bacterial systems is further highlighted by the conserved evolution of dedicated zinc homeostatic pathways, which ensure optimal zinc levels for metabolism [31,35,36,37,38].

Zinc homeostasis in bacteria: The biological consequences of zinc pose a fundamental challenge, requiring bacteria to precisely balance zinc concentrations to maintain adequate intracellular levels that sustain vital cellular functions while preventing excessive accumulation and toxicity [39,40]. The need to balance and maintain intracellular zinc levels led to the evolution of sophisticated influx, distribution, and efflux systems that regulate zinc homeostasis [39,40]. In prokaryotes, zinc homeostasis is regulated by three interconnected pathways: influx systems that take up zinc from the extracellular environment, efflux systems that export zinc to prevent zinc toxicity, and regulatory networks that monitor intracellular zinc concentrations to modulate zinc transporter expression [34,41]. High-affinity ATP-binding cassette (ABC) transporters such as ZnuABC and low-affinity ZIP family permeases facilitate zinc uptake [39]. P-type ATPases, such as ZntA, cation diffusion facilitators (CDF), and resistance-nodulation-division (RND) family pumps, facilitate zinc efflux [42].

The expressions of zinc influx and efflux systems are often controlled by zinc-regulated metalloprotein transcription factors [33,34,35]. Examples of these zinc-responsive transcriptional repressors include Zur (zinc uptake regulator, Fur family), AdcR (MarR/SlyA family), and SmtB (ArsR family), while examples of zinc-responsive transcriptional activators include: ZntR (MerR family), ArsR/SmtB families, and SczA [34,37,43,44,45,46,47,48]. Interestingly, and unique to mycobacteria, SmtB and Zur are encoded within a single operon, whereas most bacteria encode them separately [44]. Zur and SmtB diametrically oppose each other. While zinc-bound Zur binds to the promoter regions of its target genes and represses the transcription of zinc influx systems, zinc-unbound SmtB binds to the promoter regions of its target genes and represses transcription of zinc efflux systems [44]. Thus, Zur acts as a transcription repressor under zinc-replete conditions while SmtB acts as a repressor under zinc-limited conditions [43,44].

Zinc-responsive transcriptional repressors such as Zur and AdcR bind zinc ions and, in their fully metalated and hemimetalated states, inhibit transcription of their respective zinc uptake systems by binding to promoter regions and repressing transcription [37,43,44,47,49]. The region in which Zur binds is known as the *zurbox* and consists of a conserved AT-rich region of 18–30 base pairs [50,51]. During zinc limitation, zinc-free Zur and AdcR lose their affinity for their respective DNA sequences, leading to de-repression and robust transcription of their respective zinc uptake systems [43,44,47,49].

Conversely, transcriptional activators upregulate zinc efflux or storage systems to mitigate toxicity from excess zinc [43,44,52]. For example, SczA in *S. pneumoniae* binds upstream of a palindromic DNA sequence when zinc is limited, thereby blocking RNA transcription of the *czcD* zinc exporter [37,38]. When bound to zinc, SczA binds to a distinct region upstream of the *czcD promoter*, inducing its transcription [37,38]. Another efflux pump activator is ZntR, which binds to DNA in both the presence and absence of zinc [43,48,53]. The binding of zinc by ZntR induces a conformational change in the DNA double helix, enabling access for RNA polymerase and robust transcription of the P-type ATPase and efflux pump, ZntA [43,48,53].

Although the total cellular zinc concentration is estimated to be in the millimolar range, studies in *E. coli* have determined that changes in femtomolar concentrations of free zinc can trigger either Zur-mediated zinc uptake or ZntR-dependent zinc efflux [43,44,54].

To combat zinc toxicity, bacteria utilize three main types of efflux pumps: P-type ATPases, CDF (cation diffusion facilitators) proteins, or RND (resistance and nodulation) tripartite efflux pumps [34,38,43,44,55,56]. Furthermore, P-type ATPases can be divided into two subgroups: the monovalent Cu^+^ and Ag^+^ subgroup, and the divalent Zn^2+^, Cd^2+^, Pb^2+^ subgroup [55]. Of the multiple P-type ATPases encoded by *M. tuberculosis*, CtpC (Rv3270) is the only characterized zinc-efflux pump; it is induced by high zinc concentration to confer resistance against zinc poisoning, and requires a metallochaperone, PacL (Rv3269), for its activity [57,58]. However, the transcription regulator of CtpC and PacL is yet to be identified.

To increase intracellular zinc concentrations under zinc-limiting conditions, two important ABC-transporters, AdcABC and ZnuABC, are encoded by prokaryotes [30,33,38,39,44,48,49,52]. AdcABC is primarily encoded by Gram-positive bacteria [59,60], and ZnuABC by both Gram-positive and Gram-negative bacteria [61,62,63]. In the AdcABC system, AdcA is a zinc-binding extracellular subunit, AdcB is a permease, and AdcC is an ATPase [52,59]. While the AdcABC influx system has mainly been studied in the *Streptococcus* genus, the ZnuABC system has been studied in many bacterial species, including mycobacteria [34,43,47,49,55,64,65]. The ZnuABC transporter is transcriptionally regulated by Zur and comprises three proteins: the soluble ZnuA, which resides in the periplasm, and which binds and delivers zinc to the ZnuB channel, which then transports zinc into the cytoplasm, and the ZnuC ATPase that provides the energy needed to transport the zinc ions across the inner membrane [36].

*Mycobacterium smegmatis* contains two *znuABC* operons: *znuABC1* (*Msmeg_6045-6047*) as the primary zinc uptake system, and *znuABC2* (*Msmeg_6049-6051*) [44,64], which is predicted to either be a backup importer or activated at lower zinc concentrations than *znuABC1* [64]. In fact, stepwise de-repression of Zur-controlled genes in multiple phases has been observed [44,64]. Mtb contains a reduced form of *znuABC* comprising only *Rv2059* (*znuA*) and *Rv2060* (*znuB*) with only three transmembrane domains [64]. Beyond zinc influx transporters, Zur also regulates several other genes involved in zinc homeostasis [66]. In Mtb, thirty-two genes, presumably in sixteen operons, were upregulated in a *Δzur* strain [66]. Taken together, a complex hierarchical network of gene expression is dedicated to maintaining zinc homeostasis in bacteria, highlighting the importance of zinc in bacterial physiology.

Zur is the master regulator of zinc homeostasis in bacteria: Zur belongs to the Fur (ferrous uptake regulator) superfamily of transcription factors that can reversibly bind zinc, exhibiting binding sites with a femtomolar affinity for the metal [67,68,69]. The crystal structure identifies three distinct domains of Zur: an N-terminal DNA-binding domain (DB), a C-terminal dimerization domain (D), and an interdomain hinge that contains the metal-binding M-site, which binds to one zinc ion [65,67]. The D-site, on the surface of the D domain, and the C-site, at the C-terminus, are the second and third zinc-binding sites, respectively [65,67]. The C-site acts as a structural zinc-binding site, while the M- and D-sites serve as regulatory zinc sensors [65,67]. The M-site acts like an on-off switch, while the D-site modulates and fine-tunes Zur repression, leading to a stepwise de-repression of Zur-regulated genes [65,67]. Zur is constitutively dimeric, but is loosely organized in an “open” state in the absence of zinc, while zinc binding induces conformational changes into a “closed” state that enables high-affinity DNA binding [65,67,70]. Zinc-bound Zur binds to the *zurbox*, thereby obstructing RNA polymerase and functioning as a repressor [49,65,68]. The affinity of Zur for different *zurbox* sequences in the genome can be differentiated by the number of zinc atoms in the dimer: while some *zurbox* sequences remain occupied by a partially metalated Zur, others are de-repressed [71], thus allowing stepwise expression of genes during the gradual depletion of intracellular zinc [71]. This stepwise expression may expand the scope of Zur-regulated transcriptional reprogramming from binary to modular, thereby enabling bacteria to mount a broader range of responses to zinc starvation.

Zur-regulated ribosome remodeling in Mtb under moderate zinc starvation: Zinc limitation induces Zur-dependent de-repression of the ribosomal protein paralogues lacking the zinc-binding CXXC motif [24,72,73]. During ribosome assembly, these proteins, which are called C- for the lack of the motif, kinetically outcompete their C+ counterparts with the CXXC motif, thereby remodeling the ribosome from C+ to C- composition. Overall, 7 of the 54 core ribosomal proteins possess C+/C- paralogue pairs in bacteria [72]. These include S14, S18, L28, L31, L32, L33, and L36, with each C+/C- paralogue pair sharing 30–60% protein sequence identity [67,72]. *Streptomyces coelicolor* has previously been shown to contain all seven paralogous pairs [74]. Studies in *S. coelicolor and Bacillus subtilis* demonstrate step-wise de-repression of C- ribosomal paralogues [49,65,71,75]. In *S. coelicolor*, using binding kinetics studies, Shin et al. determined that Zur had a greater binding affinity for the promoter of the *l33c-* gene than the *l32c-* gene [49,65]. Previous work in *B. subtilis* reported three distinct de-repression stages of zinc-regulated ribosomal protein paralogues, L31, L33, and S14 [75]. The hemi-metalated form Zur_2_:Zn_3_ de-represses *l31C-* and *l33C-* in the first stage, followed by *znuABC*, and the metallochaperone *yciC* in the second stage [75]. Lastly, the fully unmetalated Zur_2_:Zn_2_ results in the de-repression of *s14C-* [75]. Importantly, S14C- incorporation requires de novo ribosome assembly as it is an essential factor for ribosome biogenesis [75].

The genes encoding C- ribosomal proteins in mycobacteria are usually organized in Zur-regulated operons [22,23,24,25]. While *M. smegmatis* and its closely related rapidly growing mycobacteria encode five C+/C- paralogues (S18, S14, L33, L28, and L31) [22,24,25], Mtb encodes four such pairs, S18, S14, L33, and L28 [23,24]. Notably, Mtb seems to possess a lineage-specific duplication of L28C-, thus potentially encoding two L28 C- ribosomal proteins for a single C+ paralogue [21,23,24], although only one of these (encoded by Rv2058c) is usually detected in the C- ribosomes isolated from most in vitro zinc-starved Mtb cultures. The mycobacterial C+/C- paralogues exhibit 35–60% amino acid sequence identity [22,24,25], implying that their substitutions could potentially impact the structure and function of the ribosome [28].

Multiple independent studies have demonstrated that Mtb and *M. smegmatis* respond to zinc starvation by inducing transcription of C- ribosomal proteins in a Zur-dependent manner [22,23,24,25,28], and mutations in the encoding genes lead to growth impairment under zinc starvation [22,25], indicating that ribosome remodeling serves as an adaptive mechanism of zinc homeostasis in mycobacteria—similar to the findings in *B. subtilis* [21,22,23,24,25,66,76,77]. Furthermore, Prisic et al. demonstrated that the addition of calprotectin—a metal-chelating agent released by neutrophils in necrotic granulomas—to Mtb growth media enhances the expression of C- ribosomal proteins, suggesting that Mtb bacilli residing within the extracellular environment of neutrophil-rich necrotic granulomas likely induce C-ribosomes, while those residing intracellularly within zinc-rich phagosomes of macrophages predominantly produce C+ ribosomes [23,24,25]. Consistent with this model, infection of C57BL/6 mouse with a strain of Mtb expressing a fluorescent reporter from the promoter of the C- ribosomal protein exhibited reporter expression in ~60–70% of bacilli at 14 weeks post-infection, while only 10% of bacilli expressed the reporter at 4 weeks post-infection when cells were predominantly replicating intracellularly [25]. Interestingly, Mtb cells cultured in zinc-limiting conditions appear to be more virulent than their zinc-rich counterparts [23]. Moreover, transcriptomic analyses of sputum from tuberculosis patients have illustrated zinc-starvation responses in Mtb bacilli, suggesting a zinc-limiting microenvironment in the hosts [30,78]. Taken together, these findings offer compelling evidence for zinc-responsive ribosome remodeling as a key adaptive mechanism in Mtb during its growth in vivo and possibly during transmission [78].

Ribosome hibernation is induced by zinc-starvation in mycobacteria: In zinc-starved mycobacteria, single-particle cryo-EM structures at 3.3 Å have revealed that the C- ribosome is the preferred target of the protein Y homolog called Mpy (mycobacterial protein Y), which binds to the decoding center on the 30S subunit and inactivates the 70S C- ribosomes into hibernating monosomes [25,26,27].

Ribosome hibernation in bacteria refers to the stress-induced reversible inactivation of bacterial ribosomes, in which active 70S are converted into inactive 100S dimers or 70S monosomes [79,80,81,82,83,84,85,86,87,88,89,90,91]. This process employs various specific factors, of which RMF, short and long HPF, and protein Y homologs are the most widely studied candidates and bind to the decoding center of the ribosome [79,81,82,83,84,85,86,87,88,89,90,91]. More recently, new atypical members of ribosome hibernation factors called Balon or Ribosome Tunnel Occlusion Factor (RTOF) have also been discovered, which bind either to the vacant A-site or to the nascent polypeptide exit tunnel (NPET), respectively [85,92]. Ribosome hibernation is highly conserved across the bacterial kingdom and has also been observed in eukaryotes [68,69,70,71,72,73,74,75,76,77,78]. The process of hibernation is hypothesized to preserve the cytosolic pool of associated ribosomes by preventing subunit dissociation caused by unfolded proteins, thereby making them resistant to degradation by nucleases and facilitating the resumption of translation following periods of stress [29].

Like other members of the protein-Y family of ribosome hibernation factors, Mpy contains a unique βαβββα RNA-binding topology [25]. The N-terminus of Mpy binds to the 30S ribosomal subunit proteins S3 and S9 at the head region and interacts with the 16S rRNA helices h18, h24, h44, h31, and h34, and h28, blocking translation while the C-terminus is disordered [25,26,27]. This binding site coincides with the binding region of other pY family proteins that hibernate the ribosome [25]. Mpy inactivation of non-translating 70S C-ribosomes by binding to the decoding center helps to prevent their degradation [24,25,26].

Although Mpy is constitutively expressed, its binding to the 70S C- ribosome is facilitated by the Mpy recruitment factor (Mrf), whose transcription and post-transcriptional stability are regulated by zinc. Mrf is located within the C- operon in *M. smegmatis* (MSMEG_6069) and adjacent to the second L28 C- paralogue in Mtb (Rv0106) [24,25,26]. Mrf contains the zinc-binding CXXC motif, suggesting that its stability is controlled by zinc and that the protein is post-transcriptionally regulated in a zinc-dependent manner [26]. Zinc-bound Mrf is turned over by the Clp protease system [26]. When zinc-bound Mrf is turned over, Mpy recruitment remains low at the zinc concentration that induces its transcription and ribosome remodeling [26]. Using a CRISPRi inducible knockdown system, Li et al. demonstrated that knocking down the ClpP1P2 protease resulted in Mrf stability in zinc-replete conditions [26]. Furthermore, Ziemski et al. performed a genome-wide screening of ClpC1 targets in Mtb and determined Mrf (Rv0106) as a likely candidate of ClpC1, the chaperone associated with ClpP1P2 protease [93]. ClpS has been shown to interact with ClpC1 as an adaptor for ClpP1P2 [26,94]. Li et al. could detect Mrf-ClpS interaction only under zinc-replete conditions [26]. These findings together suggest that when zinc is abundant, zinc-bound Mrf readily interacts with ClpS-ClpC1 and is degraded by the ClpP1P2 protease system, presumably through ClpC1 [26]. In zinc-starved conditions, a zinc-free form of Mrf no longer interacts with the ClpP1P2 protease system, thereby stabilizing the protein and inducing Mpy recruitment [26].

Taken together, the adaptive responses induced by zinc-starvation, including ribosome remodeling and hibernation, likely impact the physiology and metabolism in Mtb in ways that could change the growth and/or drug sensitivity of the pathogen. However, the relevance of these changes in a host depends on the extent to which zinc is sequestered from Mtb during infection.

### 1.2. Zinc Transport and Immune Regulation by Host

Like bacteria, zinc has a critical role in human biology: approximately 10% of the human proteome, including essential enzymes and transcription factors, binds zinc [95,96]. Zinc deficiency has a detrimental effect on the host’s ability to control respiratory infections [97,98,99], and it is understood that zinc is a necessity for the homeostatic maintenance and function of immune cells [97,100,101]. Broadly, inflammatory pathways including toll-like receptor activation and cytokine signaling have been shown to influence the surface expression of zinc transporters, modulating intracellular and extracellular zinc availability [102]. ZIPs or Zrt and Irt-like proteins (zinc- and iron-regulated transporters, respectively) increase cytosolic zinc by importing from extracellular and phagosomal spaces. In contrast, ZnTs (Zinc Transporters) decrease cytosolic zinc by exporting into extracellular and phagosomal spaces [102]. The modulation of both ZIP and ZnT expression in response to infection has been observed in many immune cells, including dendritic cells [103], T cells [104,105], monocytes [106,107], mast cells [108], and neutrophils. For example, bacterial lipopolysaccharide (LPS), a powerful pro-inflammatory immunological stimulator, can induce toll-like receptor activation in dendritic cells and has been shown to downregulate ZIP6 and ZIP10, thereby decreasing intracellular zinc and driving cellular maturation [103]. In contrast, ZIP8 was observed to be upregulated as a consequence of T-cell receptor activation in vitro [109]. ZIP4 mutations were also observed in systemic zinc deficiency leading to loss of Langerhans cells—epidermal macrophages—disrupting barrier function and homeostasis [110]. The effects of differential inflammatory responses to an infection result in pathway-specific zinc mobilization. Moreover, these specific responses directly affect zinc availability and storage in cell-specific instances (Table 1). While zinc bioavailability has been established as a requirement for normal immune cell function [101], it is equally important for immune effectors to limit pathogens from accessing zinc [111], and the means by which this is accomplished are extensive.

Nutritional immunity specifically refers to host strategies that restrict pathogens’ access to essential nutrients, particularly metal cofactors required for microbial metabolism and virulence [112]. Broad pro-inflammatory signaling pathways sequester zinc locally at infection sites as well as systemically [111,113,114]. For example, pro-inflammatory stimulation appears to induce blood serum secretion of the pro-inflammatory cytokine IL-6, thereby upregulating zinc importer ZIP14 (slc39A14) in hepatocytes [114]. This ZIP14 upregulation likely contributes to hepatocyte scavenging of zinc and decreased concentration of zinc in serum [114]. Moreover, intraperitoneal challenge with LPS and the subsequent systemic inflammatory response result in increased zinc uptake by ZIP14 expression in gastrointestinal cells and hepatocytes [115]. Furthermore, in comparison to the parent wild-type strain, a polymicrobial sepsis model in a ZIP14-deficient mouse produced increased levels of IL-6 and other pro-inflammatory cytokines, while reducing zinc concentration in the liver [116]. Taken together, these studies provide compelling evidence of systemic zinc sequestration during infection. Although the ultimate purpose of systemic zinc sequestration appears to be to restricts its availability to pathogens, the influence of zinc redistribution on immunological precursor cell lineage commitment likely alters the course of broader immune response.

**Table 1:** The effect of inflammation on zinc availability and lineage commitment.

Inflammatory Source	Experimental Set-Up	Target	Zinc Modulation Effect	Citations
Lipopolysaccharide (LPS); TLR4 stimulation	In vitro; in vivo	BM Dendritic Cells; Splenic CD11c + DCs	Decreased intracellular free zinc Decreased ZIP6 + 10 expression; increased Znt-1 and -4 expression.	[103]
Monocyte-derived DCs + TSST-1 superantigen; TCR stimulation	In vitro	CD4 + T Cell Receptor	3-fold increase in intracellular free zinc via ZIP6 Upregulated ZIP8 expression localized in the lysosome	[104,109]
IL-2 stimulation	In vitro	CTLL-2 Cytotoxic T Cells	Zinc concentrated in intracellular lysosomal compartments	[105]
Lipopolysaccharide (LPS)	In vitro	PBMC differentiated MΦ	15-fold increase in intracellular free zinc over 40 min	[106]
LPS-injection induced systemic IL-6	In vivo; systemic	Hepatocyte and GI cell zinc transporters	Upregulated ZIP14 on GI cells + hepatocytes during acute response; Increased zinc uptake; serum zinc reduced; Reduced ZIP upregulation in IL-6^−/−^ mice	[114,115]
Polymicrobial sepsis	In vivo	Serum cytokines; liver zinc	ZIP14^−/−^ mouse had comparatively higher pro-inflammatory cytokines (including IL-6); Lower hepatocyte zinc versus WT	[116]
Systemic LPS inflammation and G-CSF injections	In vivo; mice and humans	Common myeloid progenitors; TLR signals	Inflammatory conditions favor lineage commitment towards immunoregulatory neutrophil-like monocytes (Ym1^+^Ly6C^hi^) which suppress T-cell proliferation; expansion of classical monocytes	[117,118]
GM-CSF Supplementation	In vitro	Stimulated and infected MΦ	Significant increase in total cellular zinc; 60-fold increase in ZIP2 zinc importer; 2- and 4-fold increase in ZnT-7 and -4; GM-CSF mobilized zinc through STAT-3 and -5; reversible w/zinc supplementation	[119]

Zinc as a regulator of immune cell function: In the context of local zinc modulation at the site of infection, extracellular zinc is sequestered from both pathogens and host cells alike, primarily by the actions of activated infiltrating neutrophils (and to a lesser extent, monocytes) through the zinc-chelating heterodimer, calprotectin (S100A8/S100A9) [111,120,121]. Notably, calprotectin constitutes ~46% of all neutrophil cytosolic protein [122]. Further, neutrophils compose 50–70% of an individual’s circulating white blood cells [123]. Neutrophils extravasate into infected tissues and perform a number of first-line immune functions characteristic of the innate immune response. These include the degranulation of antimicrobial proteins and proteolytic enzymes, phagocytosing of pathogens and debris, secretion of immunomodulatory proinflammatory cytokines, and undergoing a specialized apoptotic process called NETosis [124]. Neutrophil extracellular traps (NETs) are bactericidal webs of chromatin, histones, and a variety of pro-inflammatory molecules which can be induced by a number of mechanisms, including toll-like receptors and inflammatory signals [125]. Notably, NETosis results in the release of approximately 60% of total intracellular calprotectin: ~30% in the NET complex and another ~30% into the extracellular space [126]. In addition to its ability to sequester zinc, calprotectin is known to bind other crucial transition metals including iron, copper, and manganese [127,128]. The metal sequestration properties of calprotectin, in conjunction with other S100 proteins, demonstrate their powerful immunomodulatory and microbicidal function, frequently at host–pathogen interfaces in the epithelia [129,130,131,132].

In addition to directly restricting pathogenic growth, zinc sequestration by activated neutrophils also impacts the function of other immune cells. A number of in vitro studies utilizing *N*,*N*,*N*′,*N*′-tetrakis (2-pyridinylmethyl)-1,2-ethanediamine (TPEN), a membrane-permeable zinc-selective metal chelator, examined the effects of zinc deprivation on cellular function in a variety of cells [107,114,133]. In one study, monocyte precursor cells differentiated in low-zinc conditions displayed enhanced monocyte lineage commitment [107]. TPEN altered multiple core monocyte functions specifically during differentiation, including enhanced pro-inflammatory tumor necrosis factor cytokine secretion, phagocytosis, and oxidative burst capabilities [107]. Importantly, these could be reversed by adding supplementary zinc to the culture [107]. These enhanced phagocytic and oxidative burst capabilities were subsequently observed in low-zinc human-blood-derived monocytes while in the presence of bacteria in vitro [117]. While inflammation affects zinc mobilization in the host, zinc availability and transportation in turn impacts lineage commitment, cell membrane fluidity, and the consequent immune response, thereby amplifying the modulatory function of zinc on the immune system. These effects are summarized in Table 2.

Interestingly, systemic inflammatory conditions similar to those previously described in this review have been observed to alter the differentiation of neutrophil-committed precursors towards monocytic lineage [135,136] (Table 1). While the broad comparison of in vitro TPEN-supplemented, in vitro zinc-exhausted, and in vivo systemic inflammatory conditions is imperfect, all of these models result in a similar polarization of precursor cells towards monocytic lineage commitment, underscoring the crucial role of zinc bioavailability in the modulation of this process. In contrast to enhancing the inflammatory response in monocytes and their precursors, TPEN-mediated zinc chelation appears to impair a variety of functions in human-blood-derived neutrophil granulocytes, including chemotaxis, phagocytosis, oxidative burst, degranulation, and LPS-induced pro-inflammatory cytokine secretion [133]. Multiple studies have reported that reducing the intracellular concentrations of free divalent cations, including zinc, actively prevents the induction of NETosis [137,138]. This is further substantiated by the findings that neutrophils deficient in the S100A9 protein, which forms the zinc-chelating heterodimeric protein calprotectin, were able to induce NETosis more readily [139]. This enhanced NETosis function and increased quantity of NETs, presumably due to sufficient access to zinc, enhances the macrophage phagocytosis of bacteria [139]. These studies underscore the differential responses by neutrophils and monocytes in response to pathogenic challenge, linked through the changing zinc environment in the host.

Zinc as a modulator of GM-CSF receptor signaling and myeloid cell function: Granulocyte-macrophage colony stimulating factor (GM-CSF) has long been established as a crucial regulator of functionality and proliferation of neutrophils, granulocytes and monocytes [140,141,142]. GM-CSF is produced by multiple cell types, and its receptors (abbreviated as GM-CSFR) are similarly expressed by a wide variety of cells, with the pathway inducing a variety of pleiotropic effects including upregulation of specific genes related to inflammation, survival, differentiation and cell proliferation [143]. Crucially, zinc signaling has been observed to play a key role in modulating the regulatory capabilities of GM-CSF-dependent cell differentiation and proliferation.

For example, zinc treatment of U937 leukemic pro-monocyte cells prior to their GM-CSF exposure significantly reduced the surface expression of GM-CSFR and suppressed GM-CSF/GM-CSFR-mediated STAT5 phosphorylation [118]. Similarly, a 30 min zinc treatment of human peripheral blood mononuclear cells (PBMCs) resulted in decreased surface expression of GM-CSFR on monocytes, but increased expression in neutrophils and lymphocytes [134]. When these PBMCs were incubated with GM-CSF, intracellular free zinc levels in neutrophils were significantly reduced by 15%, but a similar reduction was not observed in monocytes or lymphocytes [134]. Taken together, it appears that zinc decreases GM-CSFR and therefore STAT5 phosphorylation in monocytes—the precursor cells which may become macrophages. In inflammatory conditions, GM-CSF was able to modulate zinc transporter expression and functionality in macrophages infected with the intracellular pathogen Histoplasma capsulatum [119]. Vignesh et al. showed that GM-CSF supplementation activated intracellularly infected macrophages and significantly increased the total cellular concentration of zinc, with an approximately 60-fold increase in expression of the zinc importer ZIP2 as well as more modest 2- and 4-fold increases for zinc exporters ZnT-7 and ZnT-4, respectively [119]. Ultimately, GM-CSF was found to drive zinc mobilization through STAT5 and STAT3-mediated zinc sequestration, and resulted in enhanced oxidative burst capabilities which could be reversed upon zinc supplementation [119]. Taken together, zinc, GM-CSF, and pathogenic inflammation act in concert to dictate STAT5 phosphorylation in the monocyte/macrophage cell lineage. The precise mechanistic interplay between zinc and GM-CSF and the extent of zinc’s impact on the GM-CSFR pathway remain unclear. Further investigation of the greater effect of zinc on the GM-CSFR pathway through the zinc-modulating abilities of monocyte/macrophages may reveal novel interactions between neutrophils and monocytes at both the systemic and local levels. Overall, it is clear that inflammation leads to intracellular and extracellular zinc mobilization in the host through a panel of zinc transporters. Fundamental insights into zinc mobilization pathways and the discovery of new factors underpinning the process can be leveraged for therapeutic benefit against infections.

### 1.3. Zinc Mobilization and Neutrophils in TB Pathogenesis

Historically, macrophages have dominated conceptual frameworks of TB pathogenesis, viewed both as the primary intracellular niche for Mtb and as the structural core of granulomatous lesions [144]. However, accumulating evidence has fundamentally reshaped this macrophage-centric paradigm by revealing a central and often underappreciated role for neutrophils. As the most abundant leukocytes in human circulation and a cornerstone of the innate immune system, neutrophils mount an immediate response to microbial invasion at infection sites through an arsenal of antimicrobial mechanisms—including phagocytosis, reactive oxygen species (ROS) production, protease and antimicrobial peptide release, and the formation of NETs [145,146]. In many bacterial infections, such as those caused by *Streptococcus pneumoniae*, *Klebsiella pneumoniae*, and *Escherichia coli*, neutrophils are indispensable for pathogen clearance, and their absence results in overwhelming disease [147,148]. In TB, however, this paradigm does not readily apply. Although neutrophils are rapidly recruited to Mtb-infected lungs and frequently dominate blood transcriptional signatures [149,150], bronchoalveolar lavage samples, and histopathological sections of TB lesions [151], their antimicrobial activity rarely has a sterilizing effect on Mtb. Instead, excessive neutrophil accumulation and activation are consistently associated with lung tissue destruction, heightened inflammation, and bacterial dissemination [152,153,154]. These observations place neutrophils at the core of a central paradox in TB immunology: cells that contribute to early host defense can become potent drivers of immunopathology during persistent infection. Beyond direct antimicrobial activity, neutrophils also shape the inflammatory milieu through extensive interactions with macrophages and adaptive immune cells with profound influence on tissue pathology. Thus, the contribution of neutrophils to TB pathogenesis is multifaceted.

In the context of this review, we will limit our discussion on the emerging role of neutrophils in zinc sequestration as a component of nutritional immunity during TB pathogenesis. Neutrophils play a central role in this process through the release of metal-binding proteins and specialized granule components, with the previously introduced heterodimeric protein calprotectin at the forefront [111,120,121]. Owing to the sheer quantity of circulating neutrophils in the blood [123], and the abundant reservoir of calprotectin which again makes up half of the neutrophil cytosolic protein [122], nutritional immunity can be observed to be a highly conserved tool which is effective against a variety of pathogens. However, Mtb has evolved sophisticated mechanisms to withstand nutrient deprivation while exploiting hostile inflammatory environments.

Crucially, neutrophils contain a highly organized system of cytoplasmic granules and vesicles generated sequentially during granulopoiesis which are released in a tightly regulated hierarchy upon activation [155]. This organization enables rapid antimicrobial responses while minimizing collateral tissue damage under homeostatic conditions. In TB, however, sustained neutrophil recruitment and activation disrupt this balance, leading to excessive granule release that drives lung tissue destruction, extracellular matrix degradation, and cavitary disease [156]. Highly expressed calprotectin is also associated with secondary and gelatinase granules, and is released extracellularly during neutrophil activation, degranulation, NET formation, and cell death. Elevated calprotectin levels have been detected in TB patient sputum and plasma and correlate with neutrophil-driven inflammation, lung pathology, and disease severity, underscoring its dual role in host defense and immunopathology.

Neutrophil-driven nutritional immunity against pathogens: As previously mentioned, nutritional immunity is a fundamental host defense strategy that limits pathogen access to essential nutrients, particularly transition metals such as iron, zinc, and manganese [112,157]. Because these metals serve as critical cofactors for microbial enzymes involved in metabolism, replication, and antioxidant defense, their sequestration can profoundly restrict pathogen growth.

While this review has focused extensively on zinc, iron sequestration is the best-characterized arm of nutritional immunity [158,159]. Neutrophil-derived lactoferrin binds iron with exceptionally high affinity, imposing iron starvation on invading microbes and limiting bacterial proliferation. In parallel, neutrophils secrete lipocalin-2 (also known as NGAL), which binds catecholate-type bacterial siderophores such as enterobactin, preventing pathogens from reclaiming iron through their own scavenging systems [160,161]. Together, lactoferrin and lipocalin-2 represent complementary mechanisms that restrict extracellular and siderophore-mediated iron acquisition.

Zinc and manganese limitation constitutes another critical component of neutrophil-driven nutritional immunity and is mediated primarily by calprotectin [162]. Calprotectin binds zinc and manganese with high affinity, thereby depriving pathogens of metals required for key enzymatic processes, including superoxide dismutase-mediated antioxidant defenses [157]. By withholding these metals, calprotectin weakens microbial resistance to oxidative stress and enhances susceptibility to neutrophil-derived ROS.

These mechanisms are broadly relevant across infectious diseases. Iron restriction mediated by lactoferrin and lipocalin-2 limits the growth of *Escherichia coli* and *Klebsiella pneumoniae*, while calprotectin-dependent zinc and manganese sequestration is essential for controlling *Staphylococcus aureus* infections [132,148]. Fungal pathogens such as *Candida albicans* and *Aspergillus fumigatus* are similarly constrained by metal limitation, underscoring the versatility of neutrophil-driven nutritional immunity across diverse microbial classes.

Nutritional immunity in TB is both compelling and complex. Mtb is highly adapted to the host environment and has evolved robust strategies to resist nutrient deprivation, often limiting the effectiveness of neutrophil-mediated restriction. Iron sequestration exemplifies this challenge. Although neutrophil-derived lactoferrin reduces extracellular iron availability, Mtb produces high-affinity siderophores, including mycobactin and carboxymycobactin, which enable iron acquisition even under severely restrictive conditions. Moreover, cell death within TB lesions can release iron-containing molecules such as heme and ferritin, paradoxically replenish local nutrient pools and support bacterial metabolism within necrotic granulomas. In contrast, zinc and manganese withholding by calprotectin may represent a more formidable barrier to Mtb survival. These metals are essential for Mtb enzymes involved in oxidative stress resistance and central metabolism, and their sequestration within granulomas can impair bacterial fitness. However, Mtb can counteract this pressure by upregulating high-affinity metal transport systems such as MntH, as well as by remodeling ribosomal function to reduce metal dependence [66]. Ribosome hibernation and the subsequent slow-replicating state in Mtb under severe zinc starvation, as shown in vitro [25], highlights an ongoing evolutionary arms race between host nutritional immunity and bacterial adaptation. The ability of Mtb to persist under such oppressive conditions only to later emerge from dormancy confers great advantage to survivability.

Nutritional immunity mediated through NETs adds further complexity to TB pathogenesis. NETs are decorated with granule-derived proteins, including calprotectin and lactoferrin, embedding metal sequestration directly within extracellular antimicrobial structures [163,164,165]. While NET-associated calprotectin may restrict mycobacterial growth locally, excessive NET formation in TB has been strongly linked to lung tissue damage, extracellular matrix degradation, and cavitary pathology [126]. Thus, NET-driven nutritional immunity may impose antimicrobial pressure at the cost of exacerbated immunopathology.

Beyond direct metal sequestration, neutrophil-driven metabolic effects indirectly shape TB outcomes. Arginase-1 released from tertiary granules depletes local L-arginine levels, limiting nitric oxide production by macrophages—a critical antimicrobial mechanism against Mtb [166]. Consequently, while neutrophils restrict certain nutrients required by Mtb, they may simultaneously suppress complementary host defenses, tipping the balance toward disease progression rather than bacterial clearance.

Overall, neutrophils occupy a uniquely ambivalent position in TB immunity. Equipped with an extensive granule arsenal, they contribute to antimicrobial defense and nutritional immunity, yet Mtb has evolved strategies to resist, evade, or exploit these mechanisms. Nutritional immunity exemplifies this tug-of-war: while lactoferrin, lipocalin-2, and calprotectin impose metal starvation, Mtb counters these pressures through siderophore production, metal transport systems, and metabolic adaptation. Further, neutrophil death can inadvertently replenish nutrient reservoirs.

A deeper understanding of how neutrophil nutritional immunity operates within TB lesions is therefore critical. Dissecting the balance between protective nutrient restriction and pathological inflammation may reveal new therapeutic opportunities. Strategies that enhance beneficial aspects of neutrophil nutritional immunity while limiting tissue damage could serve as valuable adjuncts to antibiotic therapy, offering fresh avenues to combat this persistent global threat.

## 2. Concluding Remarks

**Figure 1:**
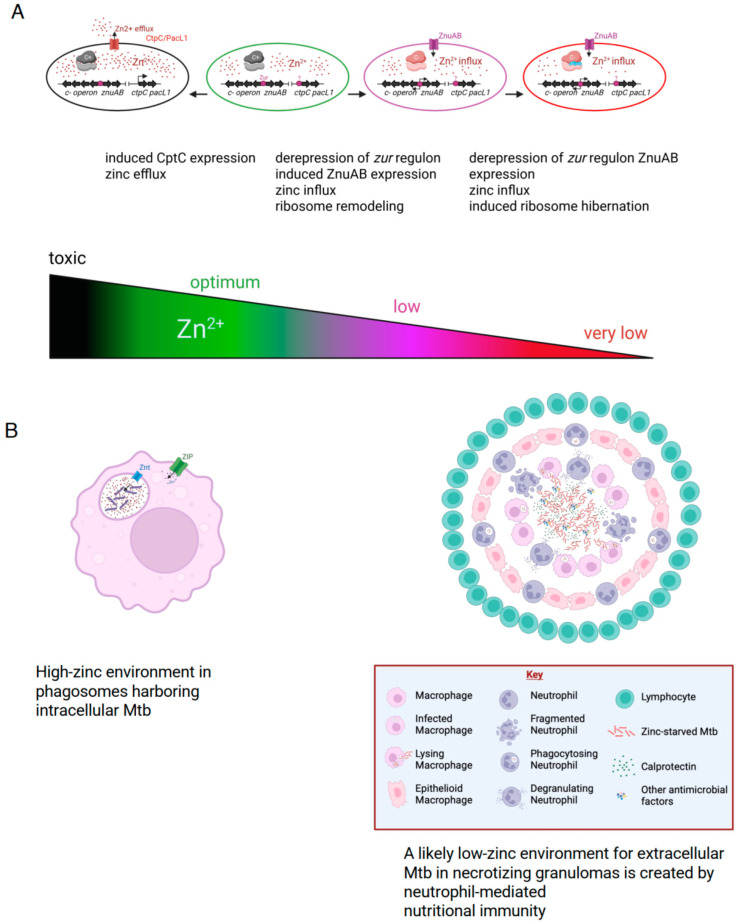
Zinc-driven host pathogen interaction in TB pathogenesis. (**A**) Adaptation of Mtb to high- and low-levels of zinc. A high zinc level, which can be potentially toxic, induces the expression of CtpC and associate chaperone PacL1. The CtpC-PacL1 likely efflux zinc out of the cytosol to mitigate toxicity (PMID: 21925112, 35961955). While a substantial free zinc is sequestered by the zinc-binding C+ ribosomal proteins in the ribosome. Under low zinc condition, derepression of Zur regulon, which includes C- rprotein paralogs and zinc importers, ZnuAB, maintain zinc level to sustain metabolic demand. Severe zinc limitation induces hibernation of C- ribosomes by Mpy, possibly to maintain a stable pool of non-translating ribosomes while also modulating the translation frequency to align with slowed metabolism. (**B**) Depiction of the high- and low-zinc environments for Mtb in the host. The high-zinc environment in the phagosomal compartments harboring intracellular Mtb is likely produced by the zinc-mobilizing activities of ZIP and Znt transporters across the plasma membrane and phagosomal membrane, respectively (PMID: 21925112). The low-zinc condition is likely presented to the extracellular Mtb bacilli in necrotizing granulomas through neutrophil-dependent nutritional immunity, in which the release of zinc chelating factors like calprotectin released by degranulating neutrophils sequester free zinc from the bacilli. The figure was created using Biorender licensed to A: Corro, J. (2026) https://BioRender.com/lky83r8, accessed on 5 February 2026, B: Corro, J. (2026) https://BioRender.com/l07yi0d, accessed on 5 February 2026.

Zinc is a crucial resource for bacteria and is necessary for metabolic activity. The sequestration of zinc from pathogens by the host’s innate immune response could be a powerful defense mechanism to restrict invading pathogens. However, the adaptation of Mtb to low-zinc environments could not only counter this host pressure, but also result in increased antibiotic tolerance or persistence (Figure 1), thereby posing significant challenges during TB chemotherapy.

Crucially, calprotectin, GM-CSF, and inflammation-mediated zinc transporter upregulation on hepatocytes appear to modulate zinc availability both locally and systemically, affecting both host and pathogen. The direct chelation and uptake of free zinc from the extracellular space to impose a zinc-deficient environment for the pathogen could also directly affect signaling in hematopoietic and inflammatory pathways. As neutrophils regulate zinc availability in the tissue microenvironment and are also affected by zinc, a deeper understanding of neutrophil-dependent nutritional immunity and its impact on TB pathogenesis may reveal new therapeutic opportunities.

Due to system-wide tight regulation of zinc homeostasis in the host, direct intrapulmonary zinc supplementation into localized sites of infection may induce therapeutic benefit when utilized in conjunction with conventional targeted therapies such as antibiotics. Such supplementation may subvert the ability of Mtb to persist in lungs. Ultimately, strategies that enhance the beneficial aspects of neutrophil-mediated nutritional immunity while limiting tissue damage could serve as valuable adjuncts to antibiotic and/or nutritional therapy, offering fresh avenues to combat this persistent global threat.

## Figures and Tables

**Table 2:** The effect of zinc modulation on immune cells.

Zinc Altering Factors	Experimental Set-Up	Target Cells	Effects	Citations
ZIP-6 overexpression	In vitro	BM Dendritic Cells	Prevented LPS-induced reduction in intracellular free zinc; MHCII upregulation	[103]
Zinc chelator TPEN	in vitro	CTLL-2 Cytotoxic T Cells	Zinc removed from zinc fingers; ERK-pathway blocked; cytoplasmic free zinc is required for IL-2-induced ERK signaling and T-cell proliferation	[105]
ZnSO_4_ supplemented cell culture (5 min)	In vitro	PBMC differentiated MΦ	Increased intracellular zinc	[106]
TPEN/zinc-depleted medium + 1α 25-dihydroxyvitamin D3 (1,25D3) monocyte differentiator	In vitro	HL-60 (pro-myelocytic) cell line	Decrease in intracellular free zinc and reduced ZIP transporter expression; increased expression of monocyte markers CD11b and CD14; TNF secretion, phagocytosis, oxidative burst were augmented by differentiation in low zinc conditions	[107,133]
Genetic ZIP-4 mutation; nonfunctional intestinal zinc importer	In vivo; humans and mice	Langerhans MΦ	Severe zinc deficiency; profound loss of skin-resident macrophages; severe dysregulation of function in NK cells, granulocytes, monocytes, and macrophages	[110]
Reduced intracellular free zinc	In vitro	Neutrophils	Low zinc availability prevents NETosis induction; S100A9^−/−^ neutrophils (non-functional calprotectin) more readily promoted NETosis	[134,135,136]
ZnSO_4_ supplemented cell culture (30 min)	In vitro	U937 leukemic pro-monocytes	Zinc alters membrane fluidity; reduced surface expression of GM-CSFR; Suppression of GM-CSF/GM-CSFR mediated STAT5 phosphorylation	[118]
ZnSO_4_ supplemented cell culture (30 min)	In vitro	Human PMBCs	Zinc decreased GM-CSFR on monocytes; increased GM-CSFR on neutrophils. Subsequent GM-CSF treatment reduced free zinc in neutrophils by 15%	[134]

## Data Availability

No new data were created or analyzed in this study. Data sharing is not applicable to this article.
